# MYL6B, a myosin light chain, promotes MDM2-mediated p53 degradation and drives HCC development

**DOI:** 10.1186/s13046-018-0693-7

**Published:** 2018-02-13

**Authors:** Xingwang Xie, Xueyan Wang, Weijia Liao, Ran Fei, Nan Wu, Xu Cong, Qian Chen, Lai Wei, Yu Wang, Hongsong Chen

**Affiliations:** 1 Peking University People’s Hospital, Peking University Hepatology Institute, Beijing Key Laboratory of Hepatitis C and Immunotherapy for Liver Disease, Beijing, 100044 China; 2 Chinese Center for Disease Control and Prevention, Beijing, 102206 China; 3 Laboratory of Hepatobiliary and Pancreatic Surgery, Affiliated Hospital of Guilin Medical University, Guilin, China

## Abstract

**Background:**

Identification of novel MDM2 or p53 binding proteins may reveal undefined oncogenes, tumor suppressors, signaling pathways and possible treatment targets.

**Methods:**

By means of immunoprecipitation and Mass Spectrometry analysis, we aimed to identify novel regulators of the MDM2-p53 pathway. We further clarified the impact of MYL6B on the p53 protein level and on the process of apoptosis. We also investigated the role of MYL6B in hepatocellular carcinoma by clone formation assay and by determining the correlation between its expression and prognosis of HCC patients.

**Results:**

We identified a novel MDM2 and p53 binding protein, MYL6B. It is a myosin light chain that could bind myosin II heavy chains to form non-muscle myosin II holoenzymes (NMII). We found that MYL6B could facilitate the binding of MDM2 to p53, which consequently promotes the ubiquitination and degradation of p53 protein. We further proved that MYL6B exerts the suppression effect on p53 as part of NMII holoenzymes because inhibiting the ATPase activity of myosin II heavy chain largely blocked this effect. We also discovered that MYL6B is overexpressed in HCC tissues and linked to the bad prognosis of HCC patients. Knocking out of MYL6B dramatically suppressed the clonogenic ability and increased the apoptosis level of HCC cell lines.

**Conclusions:**

To summary, our results demonstrate that MYL6B is a putative tumor driver gene in HCC which could promote the degradation of p53 by enhancing its’ MDM2-mediated ubiquitination.

## Background

TP53, a transcription factor, plays a pivotal tumor suppression role by inducing apoptosis, cell cycle arrest and cellular senescence, so the p53 pathway abnormalities constitute the most frequent defect in human cancers, including Hepatocellular Carcinoma [1, 2]. MDM2 is an ubiquitin ligase E3 with RING finger domain, which is critical in the ubiquitination and degradation of p53 protein [3]. Many studies have shown that the most predominant function of MDM2 is to down regulate the stabilization of p53 protein and its’ downstream pathway [4].

Given the importance of MDM2-p53 pathway in varies biology processes form early development to cellular stress response, MDM2-p53 pathway accepts redundant regulations, ranging from transcription level to post-translation regulations [1]. Various proteins could bind either MDM2 or p53 protein and some could even bind both. These MDM2 and p53-binding partners maintain appropriate p53 level by regulating Mdm2-p53 interaction, ubiquination, phosphorylation, sub-cellular location or transcription [5]. Most of these binding partners are key modulator of different biological process, including oncogenes and tumor suppressors, such as HAUSP [6], p19ARF [7] and Cyclin G [8]. Hence, identification of novel MDM2 or p53 binding proteins may reveal yet undefined oncogenes or tumor suppressors, signaling pathway and possible treatment targets.

In this study, by means of immunoprecipitation and Mass Spectrometry analysis, we aimed to identify novel MDM2-p53 pathway regulator and found a previously undefined MDM2 and p53 associated protein myosin Light Chain 6B (MYL6B), also known as MLC1SA. Myosin is a superfamily of proteins that bind to actin and act as molecular motor under the energy of ATP hydrolysis. Most myosin holoenzymes are composed of heavy chains and light chains, including essential light chain (ELC) and regulatory light chain (RLC). MYL6B is an essential light chain for non-muscle Myosin II (NMII) that involves in the control of cell adhesion, cell migration and tissue architecture, cargo transport and endocytosis [9–13]. But, in this study we found that MYL6B could bind both MDM2 and p53 protein, accelerate the p53 degradation and promote HCC development.

## Methods

### Tissue samples and cell lines

All HCC cancer tissues and adjacent non-cancerous tissues were obtained from patients who underwent surgery at the affiliated hospital of Guilin Medical University (Guilin cohort). Written informed consent was obtained from all patients in this study. The hepatocellular carcinoma cell line Huh7 (ATCC CCL-185), SK-HEP-1 (ATCC HTB-52) and the kidney carcinoma cell line HEK293T (ATCC CRL-1573) were maintained in Dulbecco’s modified Eagle medium (DMEM) supplemented with 10% fetal bovine serum (Life Technologies, Carlsbad, CA).

### RNA sequencing data of HCC tissues

RNA Sequencing (RNA-seq) data of liver hepatocellular carcinoma (LIHC) in the Cancer Genome Atlas (TCGA) project [14] were obtained from the UCSC cancer browser [15]. The RNA-seq of 417 HCC tumor tissues and 50 paired adjacent non-tumor tissues (TCGA cohort) were performed using an Illumina HiSeq 2000 RNA Sequencing platform.

### Quantitative real-time PCR

Total RNA was extracted using TRIzol Reagent (Invitrogen, USA), and 1 μg of total RNA was transcribed to cDNA using SuperScript III Reverse Transcriptase (Invitrogen, USA) according to the manufacturer’s instructions. Quantitative real time PCR (qPCR) amplification was performed in triplicate 20-μl reactions containing 0.5 μl RT product and 0.5 μM gene-specific forward and reverse primers with a LightCycler480 Instrument (Roche Diagnostic Ltd., Mannheim, Germany). The sequences of the primers used were as follows: MYL6B, 5′- GAGCCTCCAGTCGATCTCTC-3’and 5’-GCAGGAAAGTCTCAAAGTCCAC-3′; and GAPDH, 5′- CCACATCGCTCAGACACCAT -3′ and 5′- GGCAACAATATCCACTTTACCAGAGT-3′. The thermal profile consisted of 1 cycle at 95 °C for 10 min followed by 45 cycles of 15 s denaturation at 95 °C, 15 s annealing at 55 °C, and 15 s extension at 72 °C. Data was analyzed with the LightCycler 480 software (Roche), determining the threshold cycle (Ct) by the second derivative max method. Relative MYL6B mRNA expression was calculated using the ΔΔ Ct after normalization to the mRNA expression level of GAPDH.

### Plasmids and antibodies

pCMV-myc3-HDM2 (Addgene Plasmid #20935) was a gift from Yue Xiong, pcDNA3 flag p53 (Addgene plasmid # 10838) was a gift from Thomas Roberts, HA-tagged ubiquitin plasmids (WT, K48, and K63) were gift from Ted Dawson [16]. LentiCRISPRv2 was a gift from Feng Zhang (Addgene plasmid # 52961), and sgRNA specificly targeting human MYL6B (6Bsg1: ACTTTGGAGAGATCGACTGG; 6Bsg2: TTATACTTTAGAGTTCAAGG and 6Bsg3: TTCCCGTGAAGAAACCAGCA) were cloned into LentiCRISPRv2 following provider’s guide. Myc-DDK-tagged Human MYL6B ORF sequence was cloned into pCMV6 (OriGene). 3FLAG-tagged MYL6B ORF sequence was cloned into pcDNA3. Rabbit polyclone antibodies anti-p53, anti-Bax, anti-MYL6B, anti-MDM2 and mouse monoclone antibody anti-GAPDH were from Proteintech Inc. Rabbit polyclone antibody anti-ubiquitin were from Dako. Mouse monoclone anti-FLAG M2, anti-FLAG M2 Affinity Agarose Gel, and anti-c-Myc Affinity Agarose Gel was from Sigma-Aldrich.

### Immunoprecipitation and mass spectrometry

Cell lines transfected myc3-MDM2 were lysed with a lysis buffer containing 50 mM Tris–HCl, pH 7.5, 150 mM NaCl, 1% Triton X-100, 1 mM EDTA and a protease inhibitor cocktail (Roche). Cell lysates obtained from about 2 × 108 cells were incubated with anti-myc affinity gel (Sigma) in 4 °C for 3 h. After binding, the affinity gel was washed with TBS plus 0.1% Nonidet P-40. Glycine-HCL (pH 2.2) was applied to elute the myc-MDM2 protein complex. Elution was resolved on NuPAGE™ Bis-Tris Gel (Life Technologies), silver stained, and subjected to NanoLC-ESI-MS/MS analysis sequencing and data analysis (ProtTech Inc.).

For endogenous co-immunoprecipitation (Co-IP), 50 ml cell lysis obtained from about 5 × 109 Huh7 cells were incubated with appropriate primary antibodies or normal rabbit IgG at 4 °C overnight, followed by addition of Protein A/G PLUS-Agarose (Santa Cruz Biotech) for 1 h at 4 °C. The immune complexes were eluted with 0.1 M Glycine-HCl (pH 2.2) after the beads was washed with TBS. Then elution was subjected to immunoblotting with indicated antibodies.

### Immunofluorescence staining

Huh7 cells were fixed with 4% paraformaldehyde, permeabilized with 0.25% Triton X-100 and blocked with 1% BSA in PBST. Cells were stained at room temperature for 1 h with one or the mixture of two primary antibodies (from different species). After washing with PBS, cells were then incubated with one or the mixture of two secondary antibodies (from different species) at room temperature for 1 h. Finally, cells were stained with DAPI and mounted for imaging.

### Lentiviral production, infection and stable cell lines establishment

The recombinant lentiviral constructs LentiCRISPRv2, as well as assistant vectors: pVSVg (AddGene 8454) and psPAX2 (AddGene 12,260) were co-transiently transfected into HEK293T cells. Viral supernatants were collected 48 h later, centrifuged at 500 g for 5 min to remove debris, clarified by filtrated through 0.45um filter (Millipore), and then concentrated with Macrosep® Advance Centrifugal Device (100 K, PALL Corporation). Lentivirus titers were determined with the QuickTiter lentivirus titer kit (Cell Biolabs, San Diego, CA). The concentrated virus was used to infect Huh7 and SK-HEP-1 cells at an MOI of 50 with 4 μg/ml polybrene. Infected Huh7 and SK-HEP-1 cells were then subjected to puromycin selection for the establishment of stable cell lines.

### Clone formation assay

The colony formation assay was performed with Huh7 or SK-HEP-1 cells after lentivirus infection. For each group, a single-cell suspension of 1000 cells was plated in 6-well plates in triplicates and cultured in DMEM supplemented with 10% FBS for 14 days. After most of the cell clones had expanded to> 50 cells, they were fixed with 4% paraformaldehyde for 10 min, and dyed with 0.05% crystal violet for 15 min at room temperature.

### Flow cytometric-based apoptosis detection

Huh7 cells were subjected to annexin-V-FITC (eBioscience) and 7-amino-actinomycin D (7-AAD) labeling followed by fluorescence flow cytometry analysis. All annexin-V positive cells were considered apoptotic cells and included in calculation.

## Results

### MYL6B binds both MDM2 and p53 proteins in HCC cell line

**Fig. 1:**
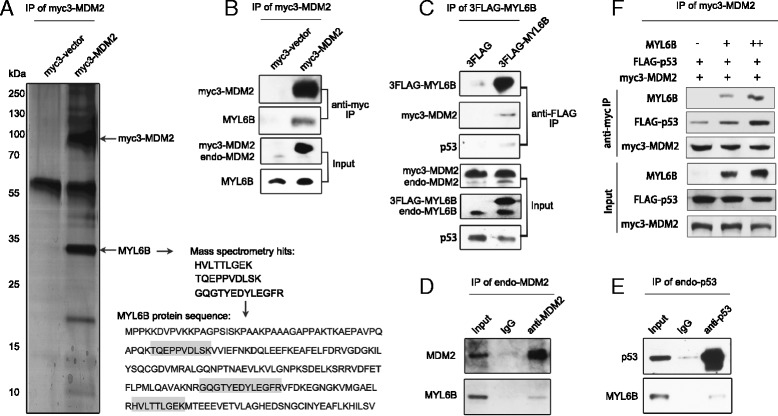
MYL6B interacts with both MDM2 and p53. (**a**, **b**) Whole cell lysate from Huh7 cell transfected with myc3-vector or myc3-MDM2 were subjected to Co-IP with anti-myc agarose and followed by NanoLC-ESI-MS/MS analysis (**a**) or immunoblotting analysis with antibody against MDM2 or MYL6B (B). (**c**) Whole cell lysate from Huh7 cell transfected with 3FLAG-vector or 3FLAG-MYL6B were subjected to Co-IP with anti-FLAG Agarose Affinity Gel and followed by immunoblotting with antibody against MYL6B, MDM2 or p53. (**d**) Huh7 cell lysate was subjected to Co-IP with anti-MDM2 or IgG and followed by immunoblotting with antibody against MYL6B. (**e**) Whole cell lysate from Huh7 cell lysate was subjected to Co-IP with anti-p53 or IgG and followed by immunoblotting with antibody against MYL6B. (**f**) Increasing amounts (0, 1, and 2 μg) of MYL6B plasmid were transfected into Huh7 together with myc3-MDM2 and FLAG-p53. Anti-myc was used to immunoprecipitate myc3-MDM2 and immunoblotting were performed with p53, MYL6B and MDM2 antibodies

Total proteins from Huh7 cells overexpressing myc3-MDM2 were subject to Co-IP and mass spectrometry analysis (NanoLC-ESI-MS/MS) in order to identify novel MDM2 binding proteins. We found that three of those highly abundant peptides hits were from MYL6B protein, suggesting that MDM2 might bind MYL6B in Huh7 (Fig. 1a). This finding was then validated by western blot analysis using a specific antibody against MYL6B (Fig. 1b).

We then overexpressed 3FLAG tagged MYL6B in Huh7, and performed Co-IP with anti-FLAG Agarose Affinity Gel to precipitate MYL6B protein and its’ binding partners. Firstly, immunoblotting analysis indicated that MDM2 was co-precipitated by MYL6B, which again supported the interaction between these two proteins. Secondly, since MDM2 and p53 are so closely regulated, we also detected if p53 could be co-precipitated by MYL6B. As expected, we found that MYL6B could also interact with p53 protein (Fig. 1c). Additionally, Co-IP of endogenous MDM2 and p53 proteins with anti-MDM2 and anti-p53 antibodies respectively in Huh7 manifested that endogenous MDM2 and p53 proteins could also interact with endogenous MYL6B (Fig. 1d, e).

As we have proved that MYL6B could bind both MDM2 and p53 proteins, we further investigated if MYL6B could enhance the interaction between MDM2 and p53. Cell lysate from Huh7 that overexpressing myc3-MDM2 was subject to Co-IP analysis by precipitating MDM2 protein with anti-myc agarose and was followed by immunoblotting with anti-p53 antibody. Co-precipitating of p53 with MDM2 protein was detectable, as have been shown in many previous studies. Interestingly, our study showed that overexpression of MYL6B significantly up-regulated the amount of p53 protein co-precipitated with MDM2 in a dose dependent manner. These data suggest that MYL6B could facilitate the binding of MDM2 to p53 (Fig. 1f).

**Fig. 2:**
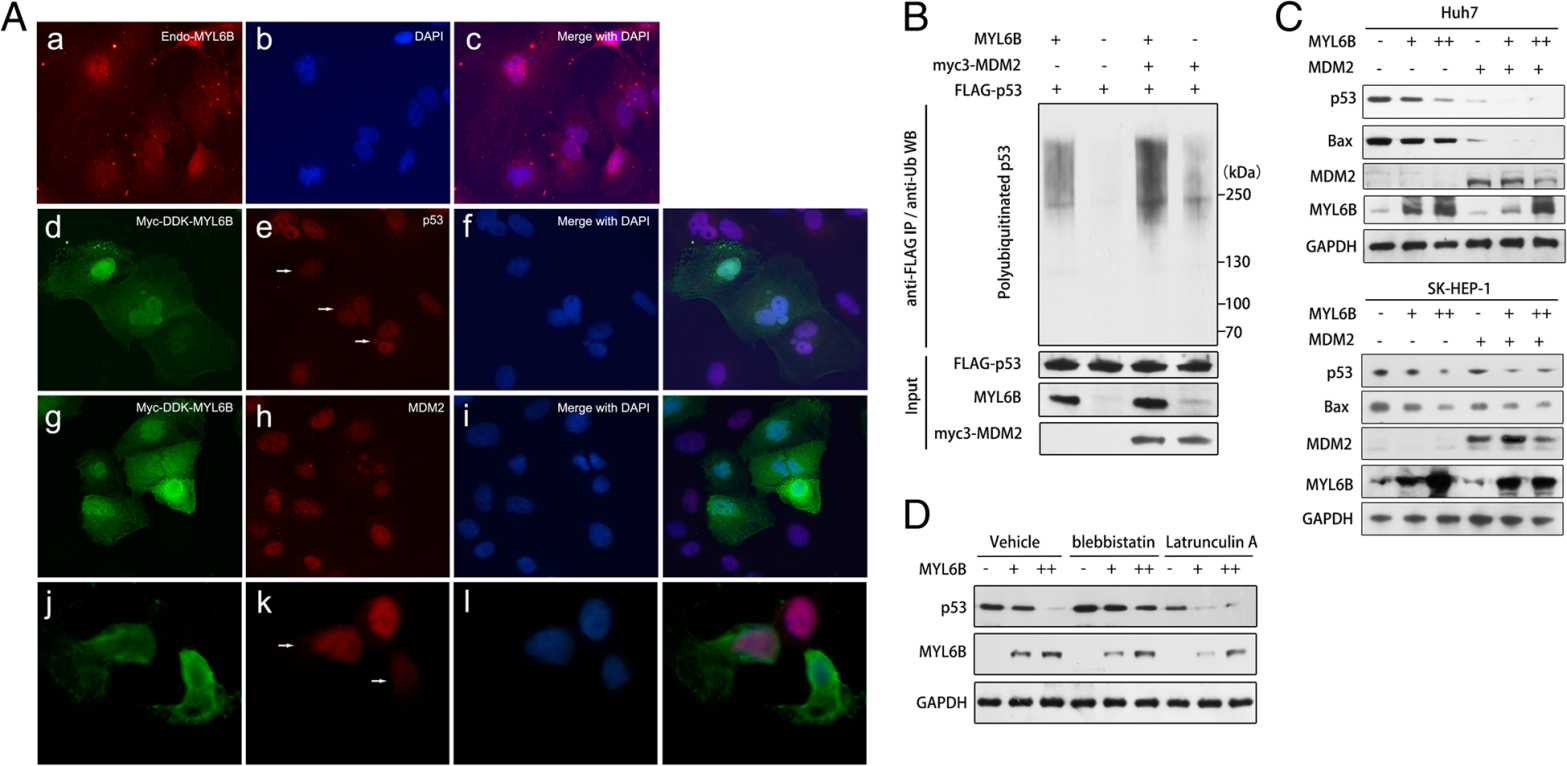
MYL6B negatively regulates p53 by promoting its’ ubiquitination. (**a**) Huh7 was processed for immunofluorescence analysis with anti-MYL6B (**a**-**c**). Huh7 transfected with Myc-DDK-MYL6B was subjected for immunofluorescence analysis with anti-FLAG and combing with either anti-p53 (d-i) or anti-MDM2 (j-l). DAPI (4′,6-diamidino-2-phenylindole) was used to label nuclei. (**b**) Huh7 was cotransfected with FLAG-p53, MYL6B and myc3-MDM2. Forty-eight hours later, cells were treated with 20 μM MG132 for 4 h and harvested for immunoprecipitation assay with anti-FLAG Agarose Affinity Gel. The eluted proteins were then subjected to immunoblot analysis with ubiquitin, p53, MYL6B and MDM2 antibodies. (**c**) Increasing amounts (0, 1, and 2 μg) of MYL6B plasmid were transfected into Huh7 (upper panel) and SK-HEP-1 (lower panel) alone or together with MDM2 plasmid, and followed by immunoblotting with p53, Bax, MDM2, MYL6B and GAPDH antibodies. (**d**) Huh7 transfected with increasing amounts (0, 1, and 2 μg) of MYL6B plasmid was treated with blebbistatin (4 μM), latrunculin A (2 μM) or vehicle and then subjected to immunoblot analysis with p53, Bax, MDM2, MYL6B and GAPDH antibodies

To assess the subcellular distribution of MYL6B in Huh7, we performed immunofluorescent staining of endogenous MYL6B and transfected Myc-DDK-MYL6B. These staining revealed that MYL6B localize in both cytoplasm and nuclear of Huh7 cells. Nuclear MYL6B colocalizes with both p53 and MDM2 as revealed by double staining. Cytoplasmic MYL6B forms grid-like filamentous network, which is consistent with the idea that myosin bind to F-actin cytoskeleton (Fig. 2a).

### MYL6B promotes p53 ubiquitination and degradation

It is well known that MDM2 is critically involved in the ubiquitination of p53. As MYL6B could enhance the interaction between MDM2 and p53, we firstly tested if MYL6B could affect the ubiquitination level of p53 protein. FLAG-tagged p53 was overexpressed in Huh7 and was immunoprecipitated. Its’ ubiquitination level was analyzed by immunoblotting with anti-ubiquitin antibody. We found that overexpressed MYL6B led to a dramatic increase in the ubiquitination level of p53 protein. When transfected together, MYL6B and MDM2 synergistically promoted the ubiquitination of p53 protein (Fig. 2b).

Since the most dominate effect of MDM2 mediated p53 ubiquitination is to promote p53 degradation through proteasome, we then investigated if MYL6B could regulate the level of endogenous p53 protein in Huh7 (Y220C/del, containing a single copy of p53 gene with Y220C). As expected, overexpression of MYL6B greatly reduced the protein level of p53 and its downstream target BAX in a dose-dependent manner in both Huh7 cells (Fig. 2c, upper panel). In order to further validate that MYL6B could negatively the protein level of wild type p53, we performed additional MYL6B overexpression experiment in SK-HEP-1 cells that contains wild type p53. These findings show that MYL6B overexpression could also negatively regulate the wild type p53 in a dose dependent manner in SK-HEP-1 (Fig. 2c, lower panel).

By immunofluorescent staining of MYL6B and p53, we found a strong negative relationship between their staining intensity. MYL6B and p53 double positive cells showed significantly decreased p53-staining intensity, which also featured by a clear dose-dependent manner (Fig. 2a-d/e/f and g/h/i). Moreover, when transfected together, MYL6B and MDM2 synergistically down-regulated the level of p53 protein (Fig. 2c). The above data manifested that MYL6B could facilitate the binding of MDM2 to p53 and consequently enhance the ubiquintation and degradation of p53 (wild type p53 and Y220C mutant p53). Although MDM2 mediated p53 ubiquitination could lead to the nuclear degradation or nuclear export of p53 [17], there is no visible co-distribution of MYL6B with p53 or MDM2 in cytoplasm, indicating that overexpression of MYL6B mainly promotes MDM2-mediated nuclear degradation of p53 (Fig. 2a-d/e/f and g/h/i).

Two previous studies have already proved that the heavy chain of non-muscle myosin II, MYH9 (NMIIA) and MYH10 (NMIIB), involved in the regulation of p53 pathway [18, 19]. As MYL6B could bind both MYH9 and MYH10 to form myosin holoenzymes [9], we further explored whether MYL6B act independent of myosin holoenzymes or act as a part of it. We found that blebbistatin mediated inhibition of myosin II’s ATPase function restricted the ability of MYL6B to negatively regulate p53 protein level, which suggesting that MYL6B exerts its p53 suppression role as part of NMII holoenzymes (Fig. 2D) [20].

Since myosins conventionally bind to F-actin, we further used latrunculin A to inhibit the assembly of F-actin and its’ association with other parterners [21]. Although actin filaments were dramatically disrupted by latrunculin A treatment, MYL6B still retained the ability to promote p53 degradation even when the F-actin polymerization was suppressed by latrunculin A treatment (Fig. 2a-j/k/l and 2D). This result suggests that MYL6B function independent of NMII’s conventional role in the process of p53 regulation.

### MYL6B overexpression correlates with bad prognosis in HCC

**Fig. 3:**
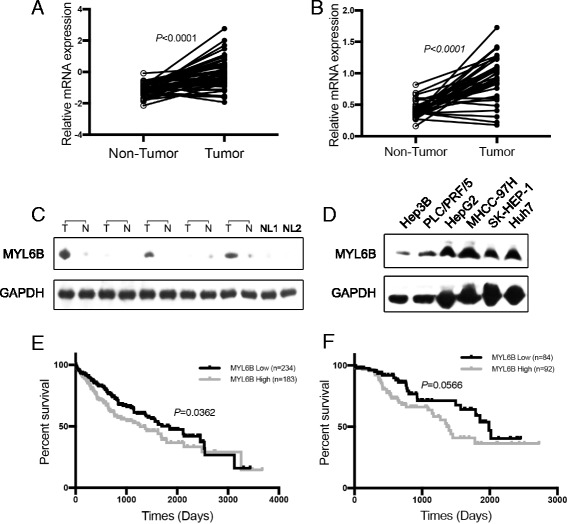
MYL6B is overexpressed in HCC and linked to bad prognosis. (**a**, **b**) Total protein extraction from HCC tissues, paired non-tumor adjacent tissues and HCC cell lineswere subject to immunoblotting with MYL6B and GAPDH antibodies. (**c**, **d**) The mRNA expression level of MYL6B in HCC tissues, paired non-tumor adjacent tissues based on RNA sequencing data (RNA-seq) from the TCGA database and its’ correlation with patient prognosis. (**e**, **f**) The mRNA expression level of MYL6B in HCC tissues, paired non-tumor adjacent tissues based on real time PCR analysis of Guilin Cohort (35 patients) and its’ correlation with patient prognosis

We have shown that MYL6B is a dominant MDM2-p53 pathway regulator in Huh7 and SK-HEP-1 cell lines, so we aimed to further investigate if MYL6B is involved in the pathogenesis process of HCC. Firstly, we found that the mRNA level of MYL6B in HCC tumor tissues is dramatically higher than in normal liver tissues according to the RNA-seq data in the TCGA LIHC dataset (Fig. 3a). The overexpression of MYL6B mRNA in HCC was further validated by QRT-PCR in an independent group of HCC patients (Guilin cohort) (Fig. 3b). Moreover, we found that MYL6B protein is frequently overexpressed in 63.3% (19/30) of HCC tumor tissues compared to adjacent non-tumor tissues and normal livers by immunoblotting analysis (Fig. 3c). We also found that MYL6B protein is highly expressed in almost all tested HCC cell lines (Fig. 3d). Most importantly, the overexpression of MYL6B mRNA is linked to the bad prognosis of HCC patients in both TCGA cohort (Fig. 3e) and Guilin cohort (Fig. 3f). Patients with higher MYL6B mRNA level tend to have shorter overall survival time.

In addition, by analysis the RNA-seq data of various tumors in TCGA project, we found that the overexpression of MYL6B mRNA was associated with the bad prognosis of patients in Acute Myeloid Leukemia (AML), melanoma, Kidney Chromophobe (KICH), Adrenocortical Cancer, Colon Adenocarcinoma and Mesothelioma (data not shown).

### MYL6B oncogene dependency in HCC cell lines

**Fig. 4:**
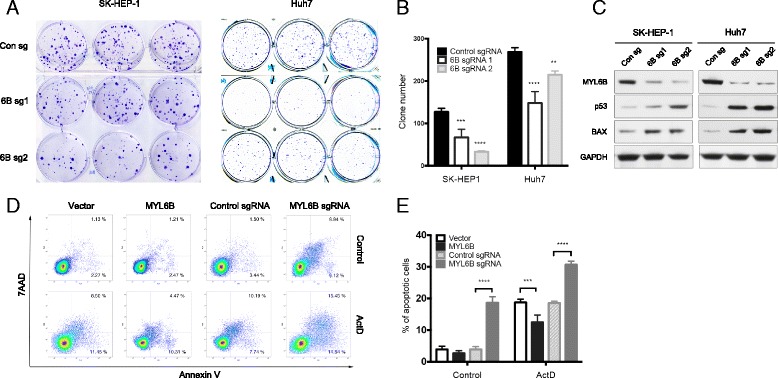
MYL6B oncogene dependency in human HCC cell lines. (**a**, **b**) SK-HEP-1 and Huh7 were infected with MYL6B specific sgRNA (6B sg1 and 6B sg2) and control sgRNA. Clone formation assay were used to test the oncogene dependency of MYL6B in both cell lines, and relative clone number was used for statistic analysis. **c** Immunoblotting was performed after MYL6B knocking out with MYL6B, p53, BAX and GAPDH antibodies. **d** After MYL6B over-expressed or knocked out in Huh7, Annexin-V and 7AAD staining was used in flow cytometry analysis to measure the apoptosis level. (**e**) The frequency of Annein-V positive cells was indicated. *p < 0.05, ***p < 0.001, ****p < 0.0001

In order to figure out if MYL6B plays a role in the development of HCC, we performed clonogenic assay to study the MYL6B oncogene dependency in HCC cell lines. We found that the clone-forming ability of both SK-HEP-1 and Huh7 cell lines was hampered by MYL6B knocking out using CRISPR/Cas9 system (Fig. 4a, b). Furthermore, dramatically elevated p53 and BAX protein was observed in both cell lines after MYL6B knocking out (Fig. 4c), which suggested that deletion of MYL6B led to the activation of p53 and its’ downstream pathway.

Because one of the most important functions of p53 and BAX pathway is to promote apoptosis, we then monitored the apoptosis level after MYL6B overexpression or deletion in Huh7. We found that silencing of MYL6B greatly increased the apoptosis level of Huh7. After actinomycin D (ActD) treatment, the DNA damage induced apoptosis could be greatly inhibited by overexpression of MYL6B, or be largely enhanced by silencing of MYL6B (Fig. 4d, e).

## Discussion

Our results provide evidence that MYL6B could bind both MDM2 and p53 proteins. We further show that MYL6B could facilitate the binding of MDM2 to p53 and consequently enhance the ubiquintation and degradation of p53 (wild type p53 and Y220C mutant p53). Although MDM2 mediated p53 ubiquitination could lead to the nuclear degradation or nuclear export of p53 [17], this study indicated that overexpression of MYL6B mainly promotes MDM2-mediated nuclear degradation of p53. Previous reports have already linked myosins to the MDM2-p53 pathway. Transcription of myosin VI is activated by DNA damage in a p53-dependent manner and possesses a prosurvival effect on cell [22]. Up-regulation of Myosin-X (Myo10) correlated with TP53 mutations and poor prognosis of breast cancer and is necessary for invasion and metastasis of mutant p53-driven cancers [23]. Myh9 have a role to stabilizing p53 protein on posttranscriptional level and its’ deactivation could promote the formation and development of Squamous Cell Carcinomas [19]. But, we for the first time reported the physical association between myosin protein and MDM2-p53, which would help to further explore the molecular mechanism behind these findings.

We found that MYL6B exerts its p53 suppression role as part of NMII holoenzymes because blebbistatin mediated inhibition of myosin II’s ATPase function restricted the ability of MYL6B to negatively regulate p53 protein level [20]. This is consistent with previous reports that both MYH9 and MYH10 are overexpressed in several tumors and linked to bad prognosis of these tumor patients [10, 16, 24–27]. But, Schramek et al. found that, in human and mouse keratinocytes, MYH9 promotes the stabilization of p53 protein and function as a tumor suppressor [19]. Yam et al. also revealed the tumor suppression potential of MYH10 in mutant p53-transformed rat embryo fibroblasts [18]. These findings suggest that non-muscle myosin may exert opposite effects on p53 dependent on the tissue context and tumor type.

We also found that MYL6B is overexpressed in HCC and is linked to the bad prognosis of patients in HCC and several other tumors. Furthermore, knocking out of MYL6B could hamper the clone-forming ability, upregulate the protein level of p53 and BAX and increase the apoptosis level in HCC cell lines. Different research groups have already reported the overexpression of MYL6B in CD133+ hematopoietic stem cell in leukemia [28], in HBV related HCC [29], in HCC cell lines [30] and correlated to the prognosis of leukemia patients [31]. Our study and all these previous reports suggest that MYL6B might have a crucial role in tumor. Taken together, these results suggested that MYL6B is a putative HCC driver gene and extensive studies should be conducted in the future to investigate the role of MYL6B in HCC and other tumors.

## Conclusions

To summary, our results demonstrate that MYL6B is a putative tumor driver gene in HCC which could promote the degradation of p53 by enhancing its’ MDM2-mediated ubiquitination.
